# Analysis of transient hypermorphic activity of *E(spl)^D^* during R8 specification

**DOI:** 10.1371/journal.pone.0186439

**Published:** 2017-10-16

**Authors:** Adam T. Majot, Ashok P. Bidwai

**Affiliations:** Department of Biology, West Virginia University, Morgantown, West Virginia, United States of America; National Institutes of Health, UNITED STATES

## Abstract

Drosophila *atonal* (*ato*) is required for the specification of founding R8 photoreceptors during retinal development. *ato* is regulated via dual eye-specific enhancers; *ato-3’* is subject to initial induction whereas *5’-ato* facilitates Notch-mediated autoregulation. Notch is further utilized to induce bHLH repressors of the *E(spl)* locus to restrict Ato from its initial broad expression to individual cells. Although Notch operates in two, distinct phases, it has remained unclear how the two phases maintain independence from one another. The difference in these two phases has attributed to the hypothesized delayed expression of E(spl). However, immunofluorescence data indicate that E(spl) are expressed during early Ato patterning, suggesting a more sophisticated underlying mechanism. To probe this mechanism, we provide evidence that although E(spl) exert no influence on *ato-3’*, E(spl) repress *5’-ato* and deletion of the *E(spl)* locus elicits precocious *5’-ato* activity. Thus, E(spl) imposes a delay to the timing in which Ato initiates autoregulation. We next sought to understand this finding in the context of *E(spl)*^*D*^, which encodes a dysregulated variant of E(spl)M8 that perturbs R8 patterning, though, as previously reported, only in conjunction with the mutant receptor *N*^*spl*^. We established a genetic interaction between *E(spl)*^*D*^ and *roughened eye* (*roe*), a known modulator of Notch signaling in retinogenesis. This link further suggests a dosage-dependence between E(spl) and the proneural activators Ato and Sens, as indicated via interaction assays in which *E(spl)*^*D*^ renders aberrant R8 patterning in conjunction with reduced proneural dosage. In total, the biphasicity of Notch signaling relies, to some degree, on the post-translational regulation of individual E(spl) members and, importantly, that post-translational regulation is likely necessary to modulate the level of E(spl) activity throughout the progression of Ato expression.

## Introduction

The *Drosophila* retina is a hexagonal array of approximately 750 ommatidia. Each ommatidium houses eight photoreceptors, of which, the R8 is the first photoreceptor to be specified. All other photoreceptors are recruited to R8s through inductive signaling. Thus, the overall structure of the eye is dependent upon the placement of R8s, which are specified at the lagging edge of the morphogenetic furrow (MF). The MF is a dorsoventral groove that forms in the retinal anlage and it advances from posterior to anterior, starting in the late-second larval instar. R8s are specified through the proneural functions of Atonal (Ato), Senseless (Sens) and the Notch pathway [1–4].

Ato expression is dependent on two enhancers located at opposite termini of the *ato* transcription unit (*5’-ato* and *ato-3’*; [5]). Ato is first induced in a broad dorsoventral stripe through the action of Hedgehog (Hh) signaling, via *ato-3’* [6]. Once expressed, Ato elicits Notch signaling [7]. Notch, in turn, facilitates activation of *5’-ato*, which expresses Ato in clusters of 10–20 cells termed intermediate groups (IGs; [5]). Subsequently, Notch modulates the transcriptional regulator Su(H), inducing repressors of the *E(spl)* locus, which consequently extinguish *ato* expression [8, 9]. Notch’s role in the MF, first as an activator of Ato, and later as a repressor, has been termed biphasic. The mechanism of delay between these two roles has not yet been fully elucidated.

The *E(spl)* locus encodes seven bHLH-Orange repressors that bear C-terminal WRPW motifs that facilitate interaction with the corepressor Groucho (Gro, [10, 11]). Genes of this locus are expressed in various subsets throughout a variety of development contexts [8, 12–14]. Additionally, repressors of this locus contain divergent C-terminal regulatory domains (CtDs). Several E(spl) members bear putative sites for post-translational modification by protein kinases that reside in their CtDs [15–17]. The most well-studied example of these, *E(spl)m8*, referred to henceforth as *m8*, encodes a phosphorylation consensus motif for the Ser/Thr kinase CK2 [18]. CK2-phosphomimetic M8 is a hyperactive repressor that, when force-expressed, interrupts Ato and Sens-regulated R8 specification. In the native state, M8-CtD occludes interaction between M8 and Ato, though such auto-inhibition is relieved upon phosphorylation [18–20]. *E(spl)*^*D*^, a dominant *m8* allele, encodes a truncated protein product, M8* [21, 22] that lacks the CtD, bypassing the role of phospho-regulation. Due to the loss of its C-terminal WRPW motif, *E(spl)*^*D*^ cannot bind Gro. Despite this, *E(spl)*^*D*^ elicits retinal patterning defects, though only in combination with the recessive *split* allele of *Notch* (*N*^*spl*^). *N*^*spl*^ disrupts eye patterning through a reduction in the number of ommatidia and a perturbation in the distribution of photoreceptor types within ommatidia [23].

Attempts to recapitulate the *N*^*spl*^;*E(spl)*^*D*^ interaction with the GAL4-UAS binary force-expression system have revealed somewhat of a paradox. In *N*^*spl*^ flies, forced-expression of M8* recapitulates the *N*^*spl*^;*E(spl)*^*D*^ phenotype, but only when M8* is expressed early in the MF, prior to the onset of *5’-ato* activity [20]. However, *ato* is not (normally) repressed in WT flies until after robust *5’-ato* activity has been established and at a time where CK2-phosphomimetic M8 has been demonstrated to function [18]. This raises two points of interest. First, E(spl) repressors may be expressed earlier in the eye development program than previously considered; second, that the peculiarities of the *N*^*spl*^ background are poorly understood regarding the mechanism as to how *E(spl)*^*D*^ exacerbates the *spl* mutant phenotype.

To address the first point, evidence suggests that genes of the *E(spl)* locus are likely co-expressed with Ato in select cells of the MF before IG formation [9]. The co-expression of a repressor and its target suggests that either the repressor may be inactive for a time or, alternately, that the repressor does not simultaneously abrogate activity on all of its target’s enhancers. In this work, we provide evidence in favor of the latter possibility—that E(spl) prevent early activation of *5’-ato* while having no discernable effect on *ato-3’* activity.

To address why *E(spl)*^*D*^ perturbs eye patterning only in combination with *N*^*spl*^, we turned to genetic interaction assays. Studies of *N*^*spl*^ reveal WT Ato and Sens within the MF [23]. Patterning aberrations do not become apparent until after passage of the MF, where R8s become lost [23]. Thus, *N*^*spl*^, in isolation, does not readily hinder the earliest stages of retinal patterning. However, modifiers of *N*^*spl*^ (aside from *E(spl)*^*D*^) impart insight into the nature of *E(spl)*^*D*^ hyperactivity. A screen for modifiers of the eye phenotype of *N*^*spl*^ revealed the zinc finger repressor *roughened eye* (*roe*) as a strong phenotypic enhancer [24]. Subsequent analyses demonstrate that Roe is required for activation of *5’*-*ato* [25, 26]. Roe expression is stimulated by Notch signaling within the MF and, in turn, Roe binds near Su(H)-responsive enhancer elements to further regulate *E(spl)* gene expression. Thus, Roe presumably attenuates the expression of E(spl) repressors in the MF, facilitating aspects of retinal patterning [26].

Ato must commit to autoregulation via the *5’-ato* enhancer to ensure robust patterning of R8s. In this work, we have explored a dynamic role for E(spl) in its repression of Ato autoregulation. We provide genetic evidence that E(spl) repressors antagonize Ato function both before *and* after IG formation. Thus, much of IG formation is driven through *ato-3’* activity, further indicating that the *ato-3’* enhancer is active until IGs fully mature. Importantly, our analysis reveals that Notch signaling within the MF is less biphasic than it is bimodal, with both the activational and repressive pathways operating in parallel. In the MF, Notch directs simultaneous activation of both *5’-ato* and E(spl) repressors. E(spl) antagonism of Ato initially prevents *5’-ato* activity. Subsequently, E(spl) are lost from cells to facilitate IG maturation, in a process that requires Roe [26]. Once mature, IGs are subject to a restoration of E(spl). Ato, which is at this time solely dependent upon 5’-ato, is repressed by E(spl). We propose that phospho-activation of select E(spl) repressors functions to allow greater repressive capacity after IG formation, when Ato is expressed at its highest levels in the eye.

## Results

### E(spl) expression evolves with changing Ato pattern

**Fig 1:**
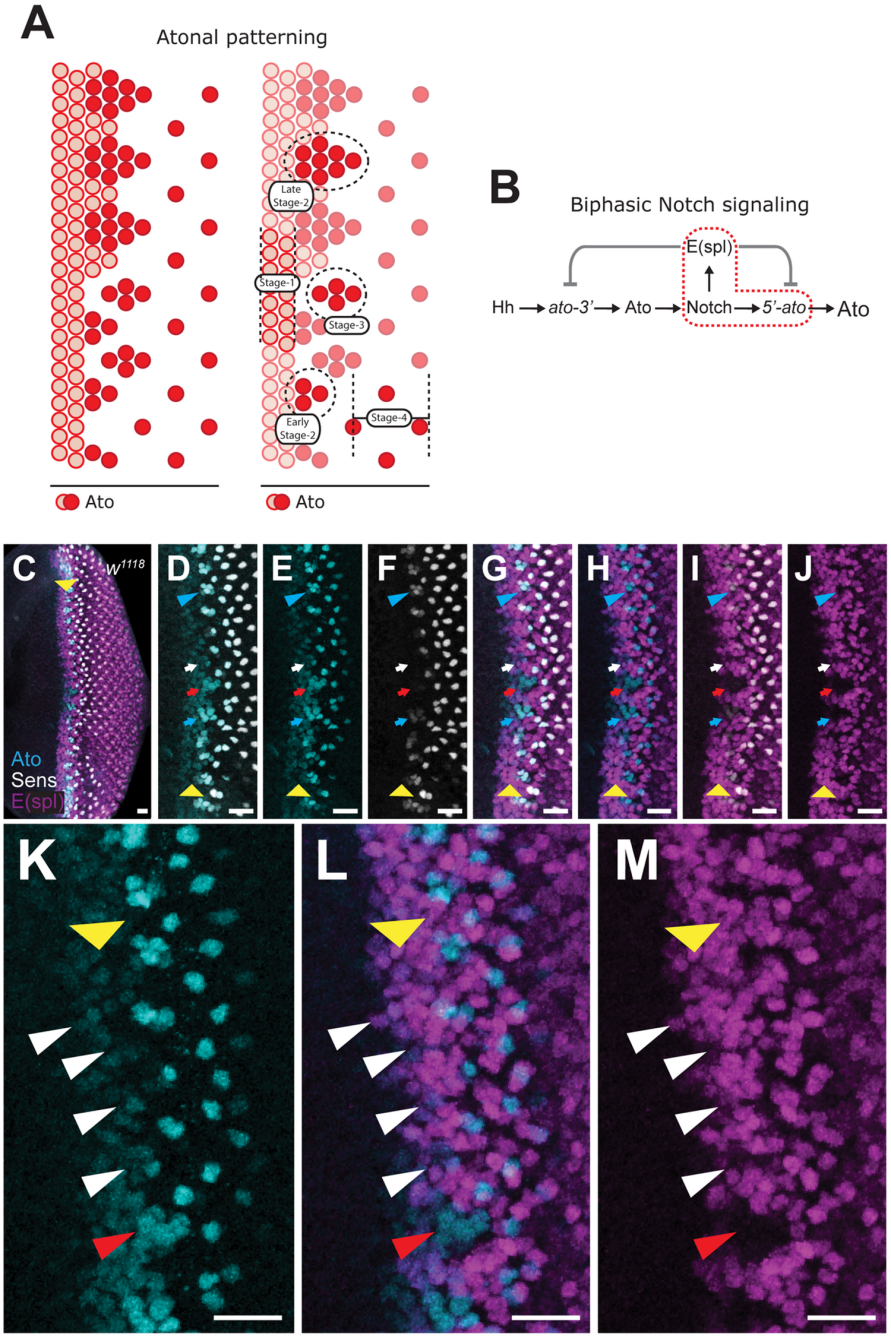
Ato colocalizes with E(spl) during early stages of R8 specification. (A) Ato expression progresses through four stages, starting at lower levels (light red) in a dorsoventral band and later is upregulated (dark red) within IGs. The latter stages are marked by repression of Ato from all cells of each IG with the exception of the R8. Here and throughout all figures, anterior is left. (B) Although Notch signaling is required to induce expression of both *5’-ato* and E(spl) repressors (inside hatching) the mechanism that separates these two activities remains unclear. (C-M) Immunostaining of WT eye-antennal discs illustrates that E(spl) (magenta) are co-expressed with Ato (cyan) during early stage-2 but not Sens (grey) in cells preceding IG formation. Yellow arrowhead denotes position of MF; scale bars = 10μm. Genotype in panels C-M is wild type (*w*^*1118*^).

Ato expression is subdivided into four stages (Fig 1). Stage-1 defines the initial induction in a broad dorsoventral band (Fig 1A). Stage-2 corresponds to the formation of IGs, which occurs contiguously with stage-1 and feature high-level Ato expression (Fig 1A, 1C–1H arrows). Throughout stage-2, Ato expression progressively increases within IGs (Fig 1D–1H, arrows). Stage-3 is reached once IGs become discontiguous from stage-2 IGs (Fig 1A, 1C–1I blue arrowhead); these cells also label for Sens, which initiates midway through IG maturation (Fig 1D–1I, arrows). Stage-4 corresponds to isolated, individual R8s (Fig 1A, 1C–1H at right). In addition to anteroposterior staging of Ato expression, IGs are also phased along the dorsoventral axis. Thus, the establishment of each IG is dorsoventrally separated by approximately 15 min. [27]. As such, a range of *ato* stages can be observed within a given sample (Fig 1A, 1C–1M).

Biphasicity of Notch during R8 specification can be summarized as the dual activation and repression of Ato (Fig 1B). E(spl), the downstream effectors of Notch, repress Ato, but it has not been clarified whether such repression affects one or both *ato* enhancers (Fig 1B, grey lines). Due to co-dependency of Ato and Notch signaling, Ato patterning can be better understood with respect to the expression of E(spl) repressors. mAb323, an antibody that recognizes several E(spl) bHLHs (mδ, mβ, mγ, m3 and, to a lesser extent, m8), reveals that E(spl) expression evolves complementary to each stage of Ato patterning [8]. At stage-1, E(spl) is undetectable due to the absence of Notch signaling at this time [7, 9]. E(spl) expression changes throughout stage-2, allowing stage-2 to be subdivided into two distinct patterns, early and late (Fig 1K–1M). Early stage-2 clusters feature both Ato and E(spl) (Fig 1K–1M, white arrowheads) whereas late stage-2 clusters are indicated by the absence of E(spl) (Fig 1K–1M, red arrowhead). By stage-3, E(spl) fully engulfs Ato-labeled clusters, which are at such point reduced to fewer cells than seen in stage 2 (Fig 1D–1J, blue arrowhead). Similarly, stage-4 R8s are fully surrounded by E(spl) labeling (Fig 1C–1M). Thus, IG maturation is marked by the progressive enhancement of Ato with the concomitant loss of E(spl); and R8 resolution is accompanied by the return of E(spl).

### E(spl) and Ato colocalize during early IG formation

Distinct IGs are first identified during early stage-2 Ato. Previous efforts using mAb323 and mAb174 (labels only E(spl)mδ) demonstrate that E(spl) co-expresses with Ato to the anterior of IGs [8, 9]. Due to improvements in microscopy technologies, a greater detail of Ato patterning can now be observed. This period of co-expression occurs as Ato is being patterned into early stage-2 IGs. This corresponds to a time when IGs are being formed, but occurs prior to their maturation into large clusters that most strongly express Ato (Fig 1). A combination of in vitro and forced-expression evidence suggests that E(spl) represses Ato function, including autoregulation [9, 28–30]. This allows the possibility that E(spl) does not globally repress Ato, as *ato-3’* is activated independently of Ato and Notch signaling.

In addition to its role in autoregulation, Ato is required for the expression of Sens [4]. Sens promotes the specification and maintenance of the R8 fate [3, 4]. Although previous reports indicate that Sens is first expressed within stage-3 clusters, our analysis indicates Sens is first induced in mature stage-2 IGs ([4, 31], Fig 1B, 1D and 1J). Sens labeling is not observed until E(spl) is fully lost from the maturing IG. This suggests that during early IG formation, E(spl) may be antagonizing Ato function (Fig 1B–1H, red arrow). Such a role is corroborated by the early upregulation of Sens in *Su(H)* mutant clones, from which E(spl) expression is lost [32].

### E(spl) delays activation of the *5’-ato* enhancer

**Fig 2:**
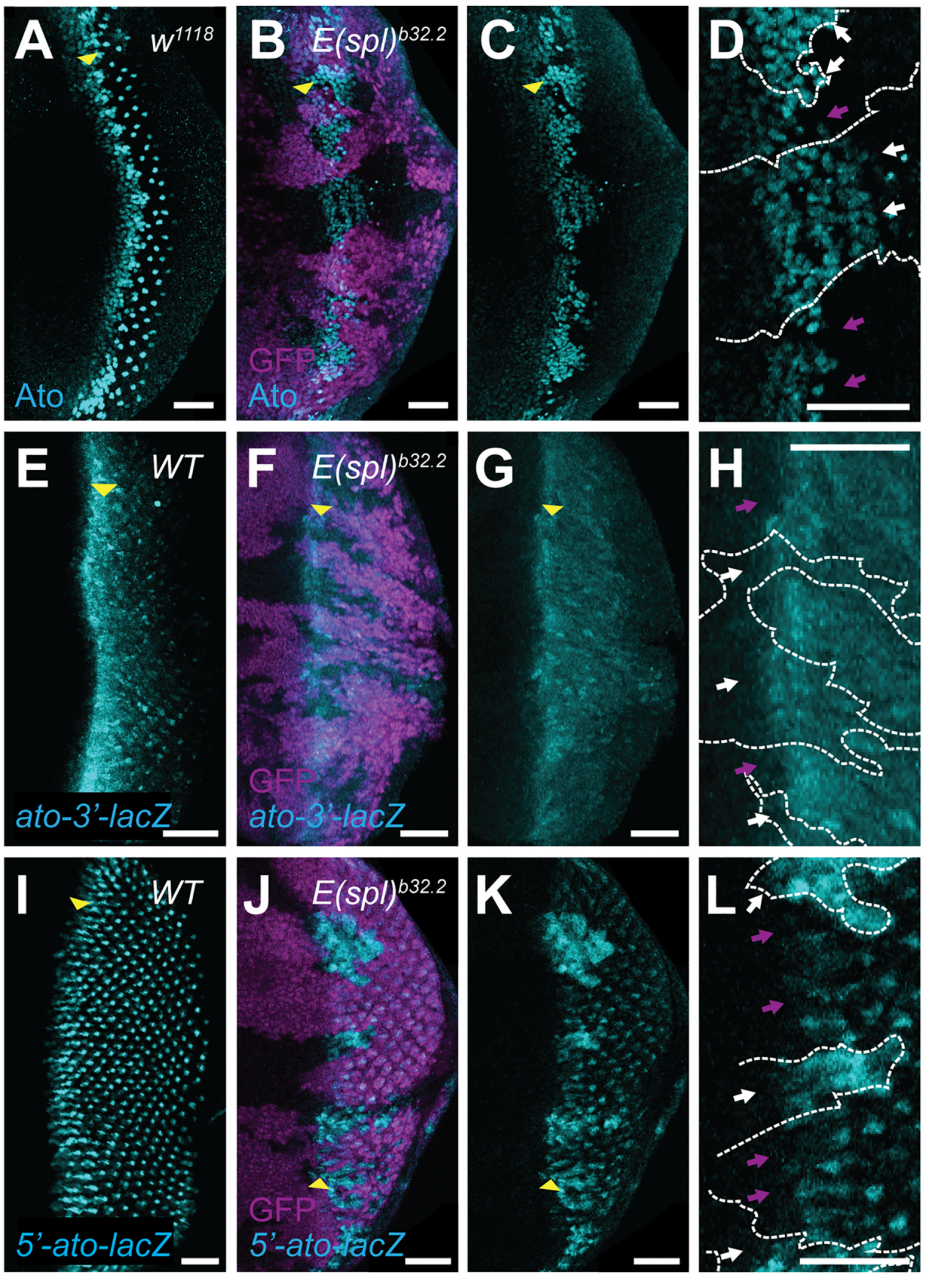
E(spl) prevents precocious *5’-ato* activation. Mitotic clones were generated using the FLP-FRT recombination system [57]. WT and heterozygous tissue is marked by GFP (magenta), mutant clones are marked by the absence of GFP. (A-D) As compared to WT eye-antennal discs (A), *E(spl)* mutant tissue features persistent, unpatterned Ato (cyan) expression. (E-H) *ato-3’–lacZ* enhancer reports in a dorsoventral band that initiates within the MF (E). Loss of *E(spl)* fails to elicit any notable effect on *ato-3’* expression (cyan). (I-L) *5-‘ato-lacZ* report mimics IG and R8 patterning of Ato, with the exception that reporter is observed in R8s throughout the posterior of the tissue (I). *E(spl)* mutants feature reporter signal (cyan) that is stronger than in WT and is observed anterior of WT or heterozygous tissues. Yellow arrowhead denotes position of MF; scale bars = 20μm. Genotypes: (A) *w*^*1118*^, (B-D) *FRT82B Df(3R)E(spl)*^*b32*.*2*^ *P{gro*^+^*}/FRT82B ubiGFP eyFLP*, (E) *P{w*^*+mC*^ *ato3’F*:*5*.*8}/+*, (F-H) *P{w*^*+mC*^ *ato3’F*:*5*.*8/+; FRT82B Df(3R)E(spl)*^*b32*.*2*^ *P{gro*^+^*}/FRT82B ubiGFP eyFLP*, (I) *P{w*^*+mC*^ *ato5’F*:*9*.*3}/+*, (J-L) *P{w*^*+mC*^ *ato5’F*:*9*.*3}/+*, *FRT82B Df(3R)E(spl)*^*b32*.*2*^ *P{gro*^+^*}/FRT82B ubiGFP eyFLP*.

*5’*-*ato* enhancer activity relies upon Ato function [5]. Thus, we sought to assess the regulatory effect that E(spl) exert on *ato* (Fig 2). As previously reported, Ato is greatly expanded in retinal tissue lacking the *E(spl)* locus, using the *E(spl)*-deficiency *E(spl)*^*b32*.*2*^ (Fig 2A–2D, white arrows; [32]). To further explore this phenotype, we examined the effect of *E(spl)* mutants on reporters for both *ato-3’* and *5’-ato*. *ato-3’* reporter expression initiates in a broad dorsoventral stripe and tapers toward the posterior margin of the eye disc (Fig 2E). Report from *ato-3’* appears unaffected in the MF of *E(spl)* mutant tissue (Fig 2F–2H, white arrows in 2H). However, *5’-ato* is greatly perturbed with respect to its WT report, exhibiting both broader expression that is not confined to IGs and an earlier report that initiates further anterior than in WT tissue (Fig 2I–2L, white arrows in 2L). This result suggests that E(spl) specifically and exclusively represses Ato through *5’-ato*, disrupting only autoregulation.

### *N*^*spl*^ enhancement by *E(spl)*^*D*^ is only moderately dosage dependent

The finding that E(spl) repress *ato* prior to IG formation may initially appear to contradict prior works that demonstrate that some E(spl) repressors require post-translational modification to repress Ato [15]. Of *Drosophila*’s seven E(spl) bHLHs, five are expressed in the MF: Mδ, Mβ, Mγ, M7 and M8 [12, 33]. Of these, Mγ, M7 and M8 are subject to C-terminal phosphorylation by protein kinase CK2; whereas Mδ and Mβ lack any apparent motif that is suggestive of C-terminal modification ([17]; Jozwick and Bidwai, unpublished). However, our data do not rule out the possibility that some repressors may be constitutively active and immediately repress Ato, whereas others are delayed in their activity.

**Fig 3:**
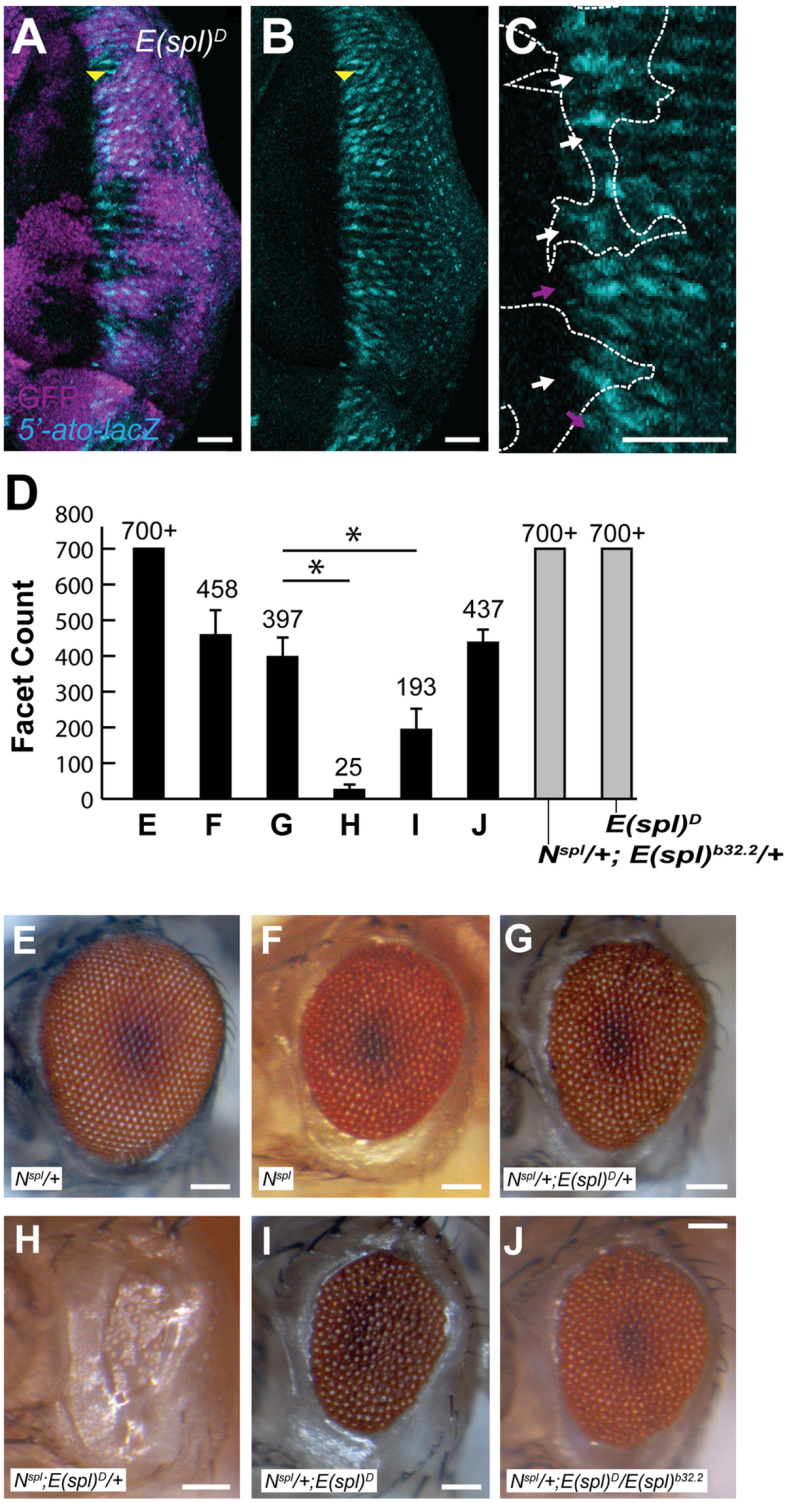
Dosage dependence of the *N*^*spl*^;*E(spl)*^*D*^ interaction. (A-C) *5’-ato-lacZ* report (cyan) is unchanged when compared between WT and *E(spl)*^*D*^ heterozygous tissue (magenta) to *E(spl)*^*D*^ homozygous tissue (lacking magenta). Yellow arrowhead denotes position of MF; scale bars = 20μm. (D) Adult facet counts for each genotype are as indicated; n≥10, asterisks denote *p-value<0.001). (E-J) Representative light micrographs of adult eyes from each genotype shown. The *N*^*spl*^; *E(spl)*^*D*^ interaction displays greater sensitivity to *N*^*spl*^ dosage than to *E(spl)* dosage. Scale bars = 100μm. Genotypes: (A-C) FRT82B e* E(spl)^*D*^; *FRT82B ubiGFP eyFLP*, (F-K) as shown.

To better assess the role of phospho-regulation of E(spl) activity, we turned to *E(spl)*^*D*^, whose protein product lacks an auto-inhibitory domain. The adult eyes of *E(spl)*^*D*^ flies are well-patterned and free of major aberration (see below). Having revealed that E(spl) represses *5’-ato*, we next tested the effect of *E(spl)*^*D*^ toward the same. Consistent with its WT adult eye phenotype, cells that are homozygous for *E(spl)*^*D*^ display no notable change in *5’-ato* activity when compared to neighboring heterozygous and WT tissue (Fig 3A–3C).

*E(spl)*^*D*^ was originally identified as a dominant modifier of the recessive allele *N*^*spl*^ [21]. *N*^*spl*^ flies bear mutant chaetae and, when homo- or hemizygous, severely reduced eyefields with aberrant retinal patterning (Fig 3F; [34]). To assess the contributions of both *N*^*spl*^ and *E(spl)*^*D*^ to this classical phenotype, we observed the effects of varied dosage. Consistent with a previous report, modulation of the defect, ranked from weakest (most similar to WT) to strongest is as follows: *N*^*spl*^/+ < *N*^*spl*^/+;*E(spl)*^*D*^/+ < *N*^*spl*^/+;*E(spl)*^*D*^ < N^spl^;*E(spl)*^*D*^/+ (Fig 3E–3H; [21]). As with *N*^*spl*^ males, which are hemizygous, homozygous females exhibit a severe, full reduction of the eye when in combination with only copy of *E(spl)*^*D*^, with few remaining ommatidia (Fig 3H). However, heterozygous females display a more limited dosage dependence on *E(spl)*^*D*^ (Fig 3G–3I). In a *N*^*spl*^/+ background, ommatidial numbers (eye size) halve upon introduction of one copy of *E(spl)*^*D*^ (*N*^*spl*^/+ compared to *N*^*spl*^/+;*E(spl)*^*D*^/+ (Fig 3D, 3E and 3G)). Ommatidial count halves again with the introduction of a second *E(spl)*^*D*^ chromosome (Fig 3D and 3I). This genetic relationship between *N*^*spl*^ and *E(spl)*^*D*^ indicates that *N*^*spl*^ contributes more greatly to the *N*^*spl*^;*E(spl)*^*D*^ interaction than does *E(spl)*^*D*^. Thus, we reason that further investigation of genetic modifiers of *N*^*spl*^ may provide further understanding of the nature of the *N*^*spl*^/+;*E(spl)*^*D*^/+ interaction, potentially providing insight into the mechanism of *E(spl)*^*D*^ hyperactivity that has, thus far, only been observed in combination with *N*^*spl*^.

### *E(spl)*^*D*^ repression is independent of *E(spl)*^*WT*^ dosage

Our analysis also indicates that *m8*, the gene primarily affected by the *E(spl)*^*D*^ lesion, is capable of repressing Ato in the absence of its Gro-binding WT allele. Increased *E(spl)*^*D*^ dosage further enhances the *N*^*spl*^ eye defect (Fig 3D, 3E, 3G and 3I). Despite strong repression in *N*^*spl*^/+;*E(spl)*^*D*^ flies, it is possible that M8* is simply eliciting hyperactivity from other E(spl) members that maintain their ability to bind Gro. To assess this possibility, we introduced the *E(spl)* deficiency chromosome, *E(spl)*^*b32*.*2*^ (with Gro rescue construct) into *N*^*spl*^/+;*E(spl)*^*D*^/+ flies (Fig 3J). Halved dosage of the entire *E(spl)* locus elicits no modulation of the eye defect in *N*^*spl*^/+;*E(spl)*^*D*^/+ flies (Fig 3D, 3G and 3J). Thus, *E(spl)*^*D*^ is acting independently of WT M8 within the MF. Furthermore, this result reaffirms that *E(spl)*^*D*^ functions independently of Gro.

### *roe* exacerbates MF mutant phenotypes

*N*^*spl*^ must be homozygous or hemizygous to completely ablate ommatidial development when in combination with *E(spl)*^*D*^. Thus, the mutant receptor is likely affecting the Notch pathway upstream of *E(spl)* to create a genetic environment that is sensitized to the specialized nature of *E(spl)*^*D*^. In a screen for modifiers of *N*^*spl*^, *roe* was identified as a strong enhancer of the *spl* eye phenotype [24]. *roe* encodes a Zn-finger repressor that is expressed in the MF [35]. *roe* is required for *ato* autoregulation, as *5’-ato* lacks any activity in the absence of *roe*, though *ato-3’* is unaffected [25]. Molecularly, Roe functions downstream of Notch signaling to suppress the expression of *E(spl)* genes [26].

**Fig 4:**
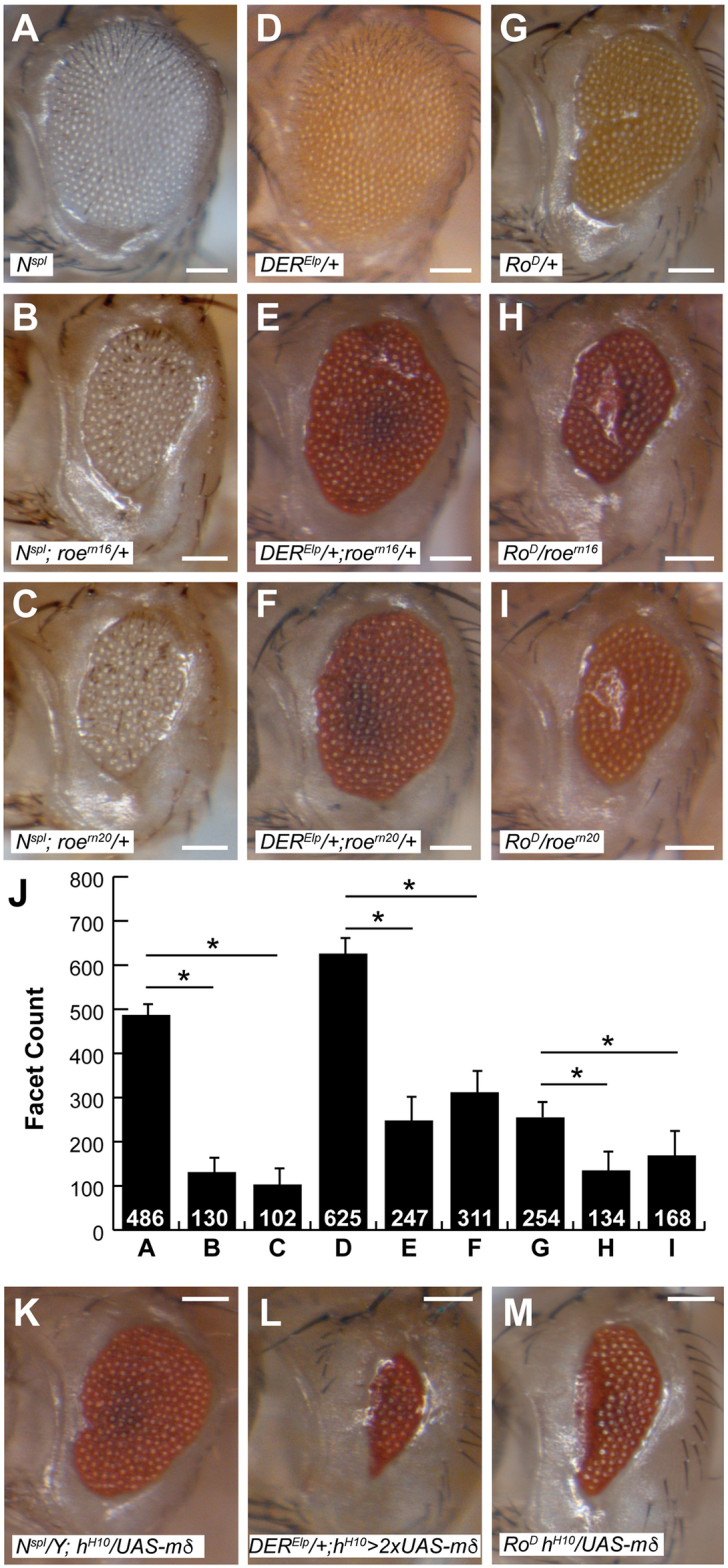
Eye mutants feature similar sensitivity to roe and E(spl) gain-of-function. (A-C, and J) *N*^*spl*^ adult eyes are sensitive to *roe*. Similarly, *DER*^*Elp*^ and *Ro*^*D*^ eyes feature enhanced reduction in combination with either allele both alleles of *roe* that were tested (D-J). (J) Eye size was quantified (facet counts) for genotypes shown in A-I; n≥10, asterisks denote *p<0.001. Additionally, all three mutant backgrounds (*N*^*spl*^, *DER*^*Elp*^, *Ro*^*D*^) were assayed for sensitivity to E(spl) gain-of-function via GAL4-driven E(spl)mδ (K-M). Qualitatively, all three backgrounds featured eyes that were further reduced in size. Scale bars = 100μm. Genotypes: (A,B,C,K) *yw N*^*spl*^ is labeled as *N*^*spl*^, all others as shown.

To further assess *roe*’s role in the MF, we quantified the ability of *roe* to modify *N*^*spl*^ and other MF-perturbed mutants (Fig 4). In agreement with previous reports, *N*^*spl*^ interacts strongly with *roe*, though only when *N*^*spl*^ is homozygous or hemizygous (Fig 4A–4C and 4J). *DER*^*Elp*^ heterozygotes feature a mildly reduced eye with rough patterning (Fig 4D; [36]). Molecularly, *DER*^*Elp*^ precociously represses *ato* and this repression is suppressed via reduced Notch signaling [28]. Ato, as affected by *DER*^*Elp*^, lacks the formation of prominent stage-2 IGs, implying perturbed *ato* autoregulation [28]. As with *N*^*spl*^, *DER*^*Elp*^ in conjunction with *roe*, exhibited a reduction in ommatidial number (Fig 4E, 4F and 4J). *Ro*^*D*^ is a dominant allele of *rough* (*ro*), which encodes a homeorepressor of *ato* that is normally expressed posterior to the MF [27, 37, 38]. *Ro*^*D*^ eyes feature a distinct anterior cleft which results from failed MF gene expression and a resultant breakdown of morphogen production (Fig 4G; [38, 39]). The *Ro*^*D*^ mutation affects the timing of Ro expression though the protein product remains unchanged [38]. Early expression of Ro, from either a heterologous promoter or the *Ro*^*D*^ mutation, elicits Ato defects [27, 38]. In combination with *Ro*^*D*^, *roe* elicits reduction in the number of ommatidia (Fig 4H, 4I and 4J). These data suggest that in each of the backgrounds assayed, *roe* contributes to further disruption of an already deficient *ato* autoregulation.

### Ectopic E(spl) also exacerbates MF mutants

**Fig 5:**
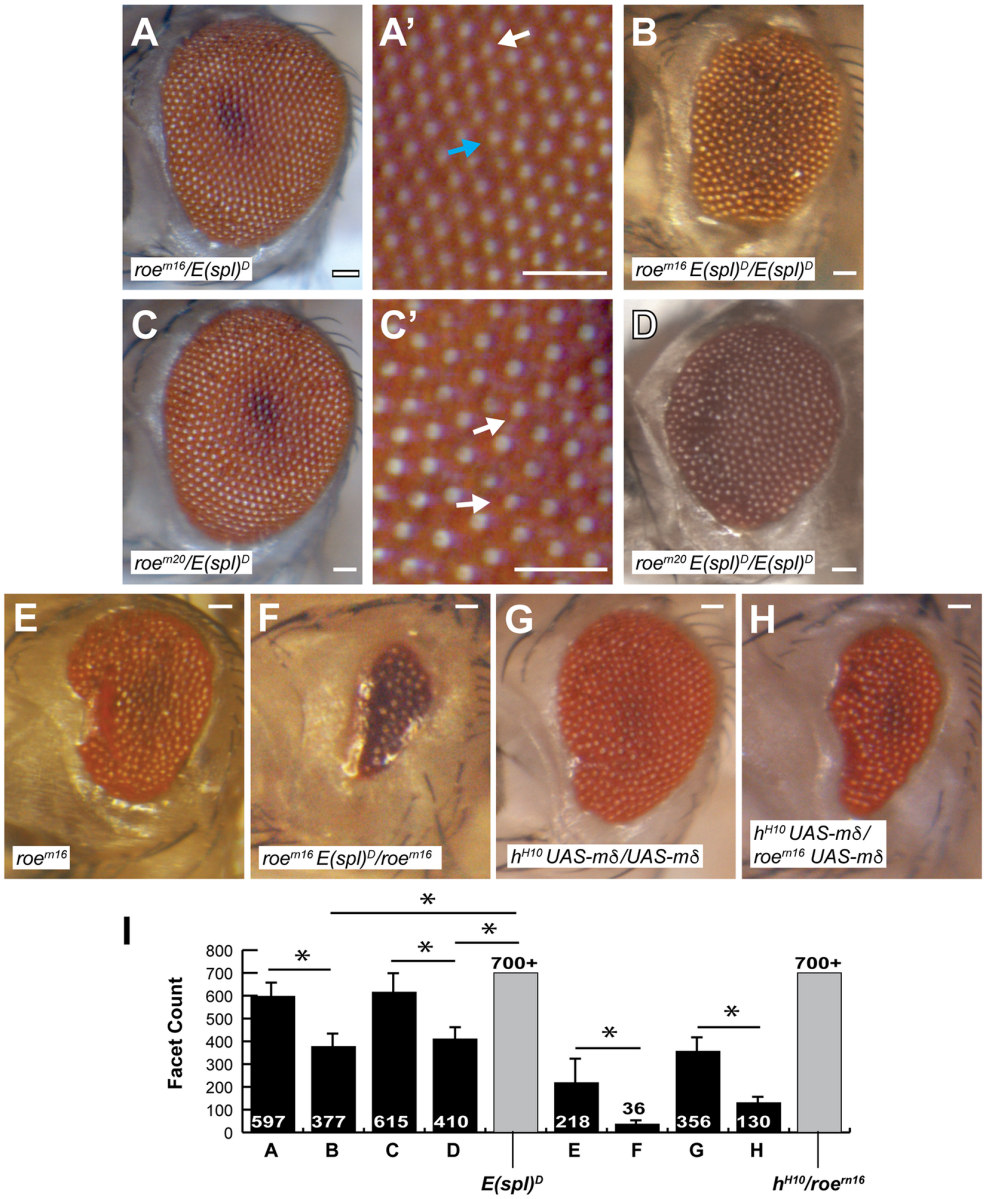
*E(spl)*^*D*^ genetically interacts with *roe*. (A) *roe*^*rn16*^/*E(spl)*^*D*^ transheterozygotes feature reduced and irregularly patterned eyes. (B) Similarly, *roe*^*rn20*^ interacts with *E(spl)*^*D*^. (C-D) The eyes of *roe* heterozygotes are further reduced when in combination with homozygous *E(spl)*^*D*^. (E-F) *E(spl)*^*D*^ severely enhances the eye defect of *roe*^*rn16*^ homozygotes. (G-H) *roe*^*rn16*^ elicits further reduction of facet count in animals that feature force-expression of two copies of *UAS-mδ*. (I) Eye size was quantified (facet counts); n≥10, asterisks denote *p<0.001. Scale bars = 50μm. Genotypes are as shown.

Of the three aforementioned MF mutants (*N*^*spl*^, *DER*^*Elp*^, *Ro*^*D*^), *E(spl)*^*D*^ interacts with only *N*^*spl*^. However, force-expression of Mδ using the Gal4-UAS binary expression system exacerbates eye defects in all three mutant backgrounds (Fig 4K–4M), serving to further correlate sensitivity of these phenotypes to both *E(spl)* gain-of-function and *roe* loss-of-function. Forced expression of Mδ with the h^H10^ GAL4 driver elicits loss of both R8s and adult ommatidia [29]. In our hands, expression of a single copy of UAS-mδ has no effect on the adult eye (Majot & Bidwai, unpublished), whereas expression of two copies elicits a loss of ommatidia and an anterior divot that is similar to the furrow-stop phenotype, as previously described (Fig 5G; [39]). This phenotype presented the opportunity to assay sensitivity of the MF mutants to increased Mδ On the basis of qualitative comparison, both *N*^*spl*^ and *Ro*^*D*^ males feature markedly smaller eyes in the presence of one copy of UAS-*mδ* (Fig 4K and 4M), whereas *DER*^*Elp*^ is sensitive to two copies of the *UAS-mδ* transgene (Fig 4L).

### *roe* interacts with *E(spl)*^*D*^

As previously shown, *roe* represses *E(spl)* within the MF [26]. We next aimed to explore a direct genetic relationship between *roe* and *E(spl)*^*D*^. As expected, *roe*, *E(spl)*^*D*^ transheterozygotes yielded perturbed and reduced adult eyes (Fig 5A, 5C and 5I). Ommatidial patterning was disrupted, with some ommatidia surrounded by only five ommatidia (Fig 5A’ and 5C’, white arrows) and others surrounded by seven or more (Fig 5A’, blue arrow). Additionally, increasing the dosage of *E(spl)*^*D*^ further enhances the eye defects (Fig 5B, 5D and 5I). To further assess this interaction, *E(spl)*^*D*^ was introduced into a homozygous *roe* mutant background. A single copy of *E(spl)*^*D*^ strongly enhances the *roe* mutant eye, vastly reducing the eye field and number of ommatidia (Fig 5E, 5F and 5I). Lastly, we asked whether *roe* loss-of-function could enhance an *E(spl)* force-expression phenotype. Force-expression of *mδ* with the *h*^*H10*^ GAL4 driver elicits a reduced eye that frequently shows an anterior cleft (Fig 5G, [29]). Reduced *roe* dosage enhances the eye defect resultant of force-expressed *mδ* (Fig 5G–5I). These data are consistent with a mechanism in which *roe* transcriptionally opposes *E(spl)* in the MF, as proposed by del Alamo and Mlodzik [26]. Our results further suggest the possibility that Roe excludes E(spl) from IGs, to permit Ato autoregulation.

### *E(spl)*^*D*^ activity is dependent upon proneural dosage

**Fig 6:**
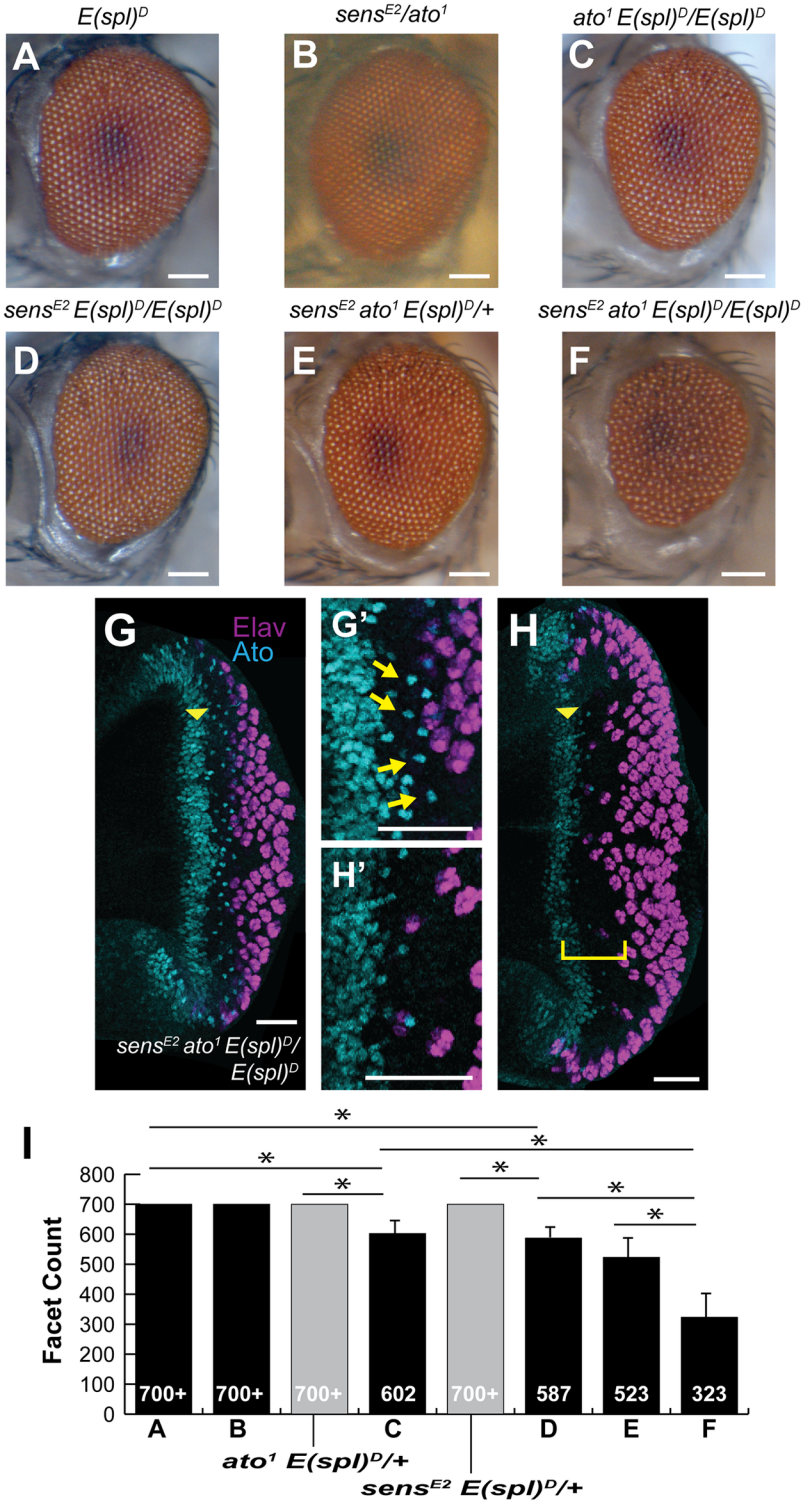
*E(spl)*^*D*^ compromises R8 specification in proneural-deficient backgrounds. (A-B) Neither *E(spl)*^*D*^ homozygotes nor *sens*/*ato* transheterozygotes feature adult eye defects. (C-D) Both *ato*^*1*^ and *sens*^*E2*^ elicit reduced eyes in combination with homozygous *E(spl)*^*D*^. (E-F) *sens*^*E2*^ and *ato*^*1*^ eyes display dosage-dependence to *E(spl)*^*D*^. (G-H) Larval retinae of the same genotype. Earlier in retinogenesis, when the MF has traversed less of the eye-antennal disc, Ato expression (cyan) is stronger with more apparent R8s (G’). As the MF progresses, individuated Ato-positive R8s become sparser (H’). Elav (magenta) immunostaining reveals defects in neural patterning. (I) Eye size was quantified (facet counts); n≥13, asterisks denote *p<0.005. Yellow arrowhead denotes position of MF; scale bars in (A-F) = 100μm and in (G,H) = 20μm. Genotypes are as shown.

Previous attempts to delineate E(spl) function in isolation of *N*^*spl*^ have made use of forced-expression approaches [18, 20, 22, 28, 29, 40, 41]. However, study of *E(spl)*^*D*^ has remained limited to its effects in the *N*^*spl*^ background [12, 20, 22]. The interaction of *N*^*spl*^/+;*E(spl)*^*D*^/+ is enhanced by the loss of the proneural genes *ato*, *da* and *sens*, and the M8 modifier *wdb* [12, 16]. However, none of these modifiers have been tested for modulation of *E(spl)*^*D*^ in an otherwise *N*^+^ background, partly due to the observation that *E(spl)*^*D*^ homozygotes elicit no major defect in eye size or patterning quality [21]. Furthermore, research into the aberrant activity of *E(spl)*^*D*^ was rendered null by the tacit possibility that *N*^*spl*^ may simply elicit a Notch signaling response in the presumptive R8. Interestingly, *ato* loss-of-function, and separately, *sens* loss-of-function, when transheterozygous with *E(spl)*^*D*^ each resulted in faint ommatidial patterning defects within the mid-posterior region of the eye field (Majot and Bidwai, unpublished). To further probe these initial findings, we observed both alleles in combination with homozygous *E(spl)*^*D*^. In both cases, the eye patterning defects became enhanced, decreasing the numbers of ommatidia and perturbing the patterning of the entire eye field (Fig 6A, 6C, 6D and 6I).

The combination of *sens* and *ato* mutants with *E(spl)*^*D*^ resulted in a similar exacerbation of patterning defects and ommatidial loss (Fig 6B, 6E and 6I). The severity of eye defect was dependent upon *E(spl)*^*D*^ dosage, as *E(spl)*^*D*^, when homozygous in this background, reduced the ommatidial count by over 35% (Fig 6F and 6I). To assess whether these defects occur from failed R8 formation, R8 loss or perhaps some other perturbation, we labeled eye discs of larvae corresponding to the mutants shown in Fig 6F for Ato and the neural marker Elav (Fig 4C). As with adult eyes, larval retinal patterning was amiss, in places lacking emergent R8s and ommatidia akin to that observed in *N*^*spl*^ (Fig 6G and 6H, [23]). Older discs exhibit a “cascade” effect such that as the MF progresses fewer R8s are specified, as indicated by the routine absence of stage-4 Ato (Fig 6G’, arrows, as compared to Fig 6H’). This effect may result from the compounding of failed neurogenesis during early retinogenesis. Neuronal loss elicits a corresponding loss of morphogens. The loss of morphogens further decreases the induction of proneural genes within the MF as eye development progresses. In agreement with such a scenario, more mature discs feature a more significant absence of neurons (Fig 6H, bracket, compared to Fig 6G’ arrows).

## Discussion

### An elucidation of the biphasic Notch signaling mechanism

The dynamic expression of Ato ensures proper, robust R8 specification. Retinal patterning is dependent upon employment of dual *ato* enhancers, with the Notch pathway incorporated into both the induction and repression of *5’-ato* [5, 9]. However, Notch regulates *5’-ato* through a bimodal mechanism wherein both Su(H)-independent and Su(H)-dependent responses initiate simultaneously rather than through a staggered response [29]. Previously, the temporal delay between the Su(H)-independent and -dependent processes had raised the question of how Notch signaling might function at this time; that perhaps Notch is engaged twice over a short span of time, or that the Su(H)-dependent response might require that a greater threshold signal intensity be achieved.

**Fig 7:**
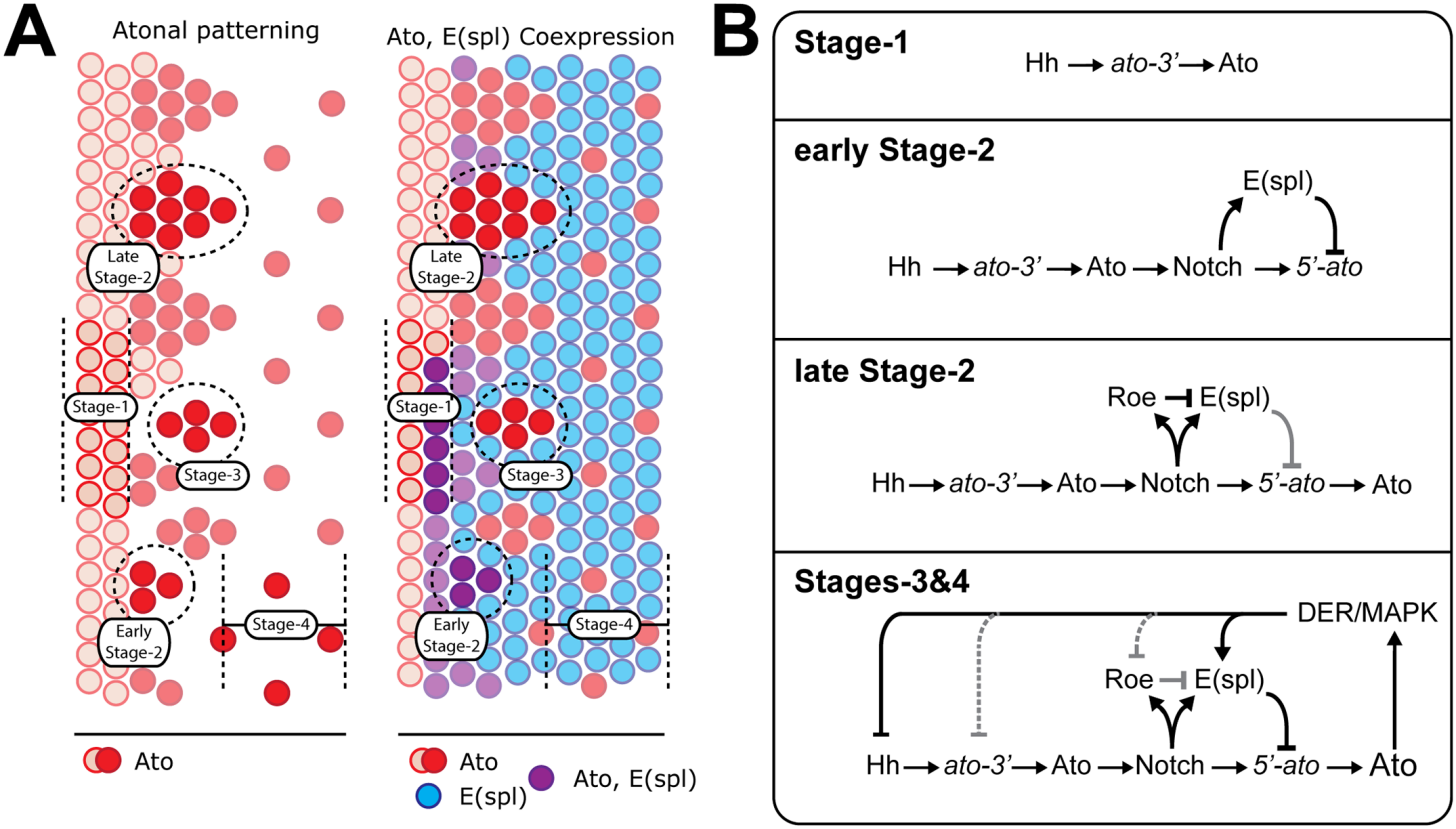
The role of E(spl) in R8 specification. (A) At left, Ato patterning as shown in Fig 1. At right, Ato patterning overlaid with corresponding E(spl) expression. E(spl) expression initiates as evidenced by result that loss of E(spl) at this stage permits precocious 5’-ato activity. By late stage-2, E(spl) is not present, and is not again observed to colocalize with Ato. (B) Stage 1: Hh drives stage-1 Ato through ato-3’. Early Stage 2: Ato, now present, drives Notch signaling, which induces both 5’-ato and E(spl). E(spl) prevents immediate 5’-ato activity. Late Stage-2: Roe specifically downregulates Su(H)-elicited genes, which include E(spl). Ato begins to accumulate. Stages 3–4: Ato elicits MAPK activation. MAPK attenuates the Hh response, activates E(spl)M8, and, although unexplored, may affect Roe activity and/or expression.

Data are not consistent with either previously proposed scenario. As indicated by E(spl) expression data, E(spl) bHLHs can be detected prior to cells’ commitment to autoregulation (Figs 1 and 7). Loss of E(spl) at this time results in the precocious activation of *5’-ato* and *sens*, both of which are dependent upon Ato function (Fig 2; [29]). Thus, in WT flies, concurrent use of both modes of Notch signaling (Su(H)-dependent and -independent) allows E(spl) to repress autoregulation (Fig 7A and 7B, early stage-2). During early stage-2, Ato expression is solely dependent upon *ato-3’* activity (Fig 7A and 7B, early stage-2). The transition from early to late stage-2 is coincident with IG maturation, during which E(spl) is lost and Ato is induced from both its 3’ and 5’ enhancers.

### A better understanding of *E(spl)D*

With this mechanism as a guide, we next sought to better understand the hypermorphic nature of *E(spl)*^*D*^. As demonstrated, E(spl) functions during early IG formation to suppress autoregulation. However, M8 is not constitutively active but requires phospho-activation by CK2 and, putatively, the DER-signal effector MAPK [17, 18, 41]. Interestingly, MAPK is active during Ato stages-3 and -4, as Ato is resolved to R8s, but not during early stage-2 [42]. This strongly suggests that M8 is specifically activated during stages-3 and -4. Unlike its full-length counterpart, M8* is constitutively active from the loss of its auto-inhibitory domain [18–20]. Despite this gain-of-function, *E(spl)*^*D*^ elicits no major patterning defect in an otherwise WT background—not at the level of the adult eye nor during Ato patterning. Thus, evidence from this and many prior investigations reveals that *E(spl)*^*D*^ only elicits retinal patterning defects in backgrounds with compromised proneural activity and potentially disrupted Ato autoregulation.

### *roe* alters E(spl) expression

Several factors help to elucidate the mechanism of interaction between *roe* and *E(spl)D*. As previously investigated, Roe is a critical regulator of IG formation, temporarily blocking expression from at least some Su(H)-responsive genes in the midst of R8 specification [25, 26]. In addition to this, *E(spl)*^*D*^ is a hypermorph that directly antagonizes Ato [12, 18]. We propose that in combination, *roe* and *E(spl)*^*D*^ create a set of conditions that 1) provide insight into the timing at which the two defined *ato* enhancers are active, and 2) are suggestive of MAPK involvement. Data support a mechanism in which E(spl) repress Ato via only *5’-ato* ([30]; Fig 2). Thus, E(spl) can only extinguish Ato where it is solely dependent on *5’-ato*, as illustrated by the colocalization of Ato and E(spl) during early stage-2 (Fig 7). IG maturation is attributable to both the 3’ and 5’ enhancers such that by the end of stage-2, *ato-3’* activity is negligible. However, *roe*, sensitizes the eye to E(spl) by failing to block Su(H) activity within IGs (Fig 7B). In this scenario, E(spl) are able to perturb proneural function as soon as *ato-3’* activity decreases. Therefore, *E(spl)*^*D*^ enables a more potent exploitation of this phenomenon. This further suggests that the modifiers of E(spl) function also have critical roles during IG maturation. Notably, as the result of increasing Ato, MAPK becomes increasingly more active in maturing IGs [42] and enables M8 function [41]. *E(spl)*^*D*^ bypasses this mechanism in which high-level Ato triggers its own repression through the establishment of sufficient DER-MAPK signaling. *roe* merely expedites this process.

### DER-MAPK represses Ato in multiple ways

It remains unclear which signals are responsible for regulating the expression and function of *roe*. Roe expression is promoted by Notch signaling, but low-level expression can be observed in *Notch* mutant clones when labeled for Roe, indicating that other forces are at play [26]. Roe is active only after *E(spl)* has initially been expressed in the MF [26]. Additionally, *DER* mutants are sensitized to *roe* mutation (Fig 4E and 4F). Functionally, Roe appears to cease its regulation of *E(spl)* once IGs mature, coincident with the same period that MAPK becomes active. It is plausible that the DER-MAPK signaling axis may be required to downregulate *roe*. The aberrant signaling of *DER*^*Elp*^ mutants can be partially rescued by a reduction in Notch signaling, indicating that cross-regulation of the two pathways may hinge about the regulation of E(spl) [36]. Despite the accumulation of evidence that MAPK enhances/activates M8 [41], it is plausible that *roe* is also regulated to some degree by DER-MAPK.

Additionally, MAPK may negatively regulate *ato-3’*, independent of either E(spl) or Roe. The *ato-3’*-*lacZ* reporter line used in this work is not suitable for determining when reporter expression ends, as β-gal is strongly perdurant in *Drosophila* tissues. However, RNA *in situ* labeling of report from *ato-3’-lacZ* reveals that the 3’ enhancer is patterned into IGs, through at least early stage-2 [5]. Thus, report from *ato-3’* ceases at the same point of eye development where MAPK becomes strongly active. Although the possibility that DER-MAPK might negatively regulate *ato-3’* has yet to be tested, it is noteworthy that MAPK activation is required to ablate the Hh signal that originally promotes *ato-3’* expression [43].

### M8 as a ratiometric antagonist of Ato?

The eye perturbation of *sens ato E(spl)*^*D*^ flies stands in stark contrast to *E(spl)*^*D*^ interactions with *N*^*spl*^ and *roe*. As indicated, we reason that *E(spl)*^*D*^ interaction with *N*^*spl*^ and *roe* are likely the result of *E(spl)*^*D*^ misexpression. In contrast, *sens ato E(spl)*^*D*^ flies are WT for both *Notch* and *roe*, allowing us to parse *E(spl)*^*D*^ function from its misexpression phenotype. The *sens ato E(spl)*^*D*^ phenotype is not due to repression by M8 during stages-3 and -4, as WT M8 is already active at that time. Therefore, *E(spl)*^*D*^ hyperactivity is of consequence during early stage-2 in *sens ato E(spl)*^*D*^ flies. Immunolabeling against Ato in such flies indicates that the quality of Ato expression and pattern continues to degrade as the MF moves further across the developing eye field (Fig 6G and 6H). However, it remains unclear whether enhanced repression by E(spl) alone might facilitate the observed phenotype. It has been demonstrated that E(spl) is at this time capable of repressing Ato-dependent activity on *5’-ato* and *sens* [32]. Though speculative, it is possible that the level of E(spl) repression during early stage-2 is tuned to permit certain Ato functions while excluding others, and that abnormally high-level E(spl) repression uniformly disrupts all Ato function.

Given that M8 has no apparent requirement for either DNA-binding or Gro-interaction, repression by M8* requires ratiometric expression commensurate with that of Ato. Thus, higher expression of M8* (compared to that of M8) and lack of auto-inhibition may combine to artificially increase the ratio of active E(spl) repressors with respect to Ato in early IG formation [22]. This also hints at why a mix of E(spl) repressors, i.e. those that require post-translational modification and those that function constitutively, may be employed in the MF. The expression of Ato is markedly lower anterior to the IG when compared to expression within the IG. Signal-mediated phosphorylation would allow for the modulation of E(spl) repressor activity without altering the expression levels of E(spl), thereby permitting tuning of repression in real-time. Ato is expressed at low levels during IG maturation, and at high levels in stages-3 and -4. Thus, the use of M8 allows MAPK to specifically enhance repression only later.

### An alternate mode of repression by bHLH-O proteins

Our analysis also provides further insight into the mode of repression by M8. Genetic evidence implicates M8* as a hypermorphic repressor (Fig 3; [22]). This enhancement exists despite M8*’s inability to bind the corepressor Gro [18, 22]. The Achaete-Scute (Asc) family of bHLH proneural activators is employed in the specification of sensory organ precursors (SOPs) during *Drosophila* peripheral neurogenesis. Similar to Ato in eye patterning, Asc are expressed in clusters and resolved to single cells via Notch-mediated expression of E(spl) (reviewed in [44]). Previous studies indicate that although Gro binding is required for E(spl) repression of Asc, DNA-binding is not [45]. Subsequent analyses in *Drosophila* and *Xenopus* indicate that E(spl) and orthologous bHLH-O repressors can forego DNA-binding by directly interacting with their proneural targets [45, 46]. This interaction essentially tethers the repressors to their targets, facilitating chromatin interaction through the DNA-binding activity of the proneural activators being targeted. Repressor-proneural interactions are likely mediated through interaction of the Orange domain of bHLH-O proteins with the transactivation domains of proneural activators and their cognate E-proteins (Da in flies; [46]). However, if Gro-interaction is required for repression by E(spl), M8* could not directly repress proneural activators.

E(spl)bHLHs are presumed to function as dimers, though it is not clear whether they might function as homo- or heterodimers [22, 47]. Thus, M8* might activate full-length E(spl) repressors through dimerization. Our analysis of the *N*^*spl*^;*E(spl)*^*D*^ interaction indicates that this is not the case. *N*^*spl*^, *E(spl)*^*D*^ transheterozygotes are enhanced by the addition of a second *E(spl)*^*D*^ chromosome (Fig 3H and 3J), demonstrating that *E(spl)*^*D*^ does not enhance the activity of full-length M8. Had *E(spl)*^*D*^ elicited hyperactivity in full-length M8, the replacement of full-length M8 with the second *E(spl)*^*D*^ chromosome would have suppressed the eye phenotype of *N*^*spl*^, *E(spl)*^*D*^ transheterozygotes. To the contrary, the second copy of *E(spl)*^*D*^ further enhanced the eye perturbations of *N*^*spl*^/+;*E(spl)*^*D*^/+. To demonstrate that *E(spl)*^*D*^ does not similarly enhance another E(spl) repressor through hetero-dimerization, we introduced the deficiency allele *E(spl)*^*b32*.*2*^ into *N*^*spl*^, *E(spl)*^*D*^ transheterozygotes. The deficiency allele had little impact on the eye phenotype, despite halving the dosage of full-length *E(spl)* and effectively decreasing the likelihood of heterodimerization between M8* and full-length E(spl) (Fig 3H and 3K). This finding demonstrates that *E(spl)*^*D*^ is likely repressing Ato through the direct interaction of *E(spl)*^*D*^ with Ato, and not indirectly through an ability to activate full length repressors that are also present. Thus, *E(spl)*^*D*^ is truly, as Nagel et al. once described, a Gro-independent hypermorph [22]. A Gro-independent mode of repression suggests that the disruption of proneural-mediated transactivation is sufficient to disrupt eye patterning. Although studies of E(spl) DNA-binding independence were assessed with SOP specification (which utilizes Asc proneural activators), it is plausible that E(spl) may target Ato independently of both DNA-binding and Gro-interaction.

In total, these studies demonstrate that in retinogenesis, E(spl) are more dynamic than previously considered, featuring fast-changing expression dynamics coupled with post-translational regulation. Despite limitations, including a lack of means of directly detecting individual E(spl) proteins or Roe, our studies combine immunohistochemical and genetic interaction approaches to demonstrate that E(spl) are active earlier, albeit incrementally, than previously considered. Although slight, this adjustment to the model of retinogenesis enables a more sophisticated understanding of the interaction between Notch signaling, Ato and R8 patterning, and will hopefully serve as the basis for future investigation into possible nodes of connectivity between Notch and DER-MAPK signaling.

## Materials and methods

### Drosophila genetics

Flies were cultured on yeast-glucose media at 24°C and maintained according to a typical diurnal schedule. *Df(3R)E(spl)*^*b32*.*2*^ removes the entire *E(spl)* locus including *gro* [48]. The inclusion *of p{gro}* rescues cell-autonomous lethality caused by deletion of *gro* [49]. *E(spl)*^*D*^ encodes M8*, an M8 truncation that lacks the CtD [50, 51]. *N*^*spl*^ encodes I578T, which alters EGF repeat 14 of the Notch extracellular domain, eliciting altered fucosylation [23]. *roe*^*rn16*^ removes the entire *roe* coding region [35]. *roe*^*rn20*^ removes the entire *rn* locus, which includes roe [35]. *Der*^*Elp*^ encodes A877T, which enhances DER sensitivity to activation [28]. *Ro*^*D*^ alters the upstream enhancer of *ro*, eliciting either enhanced or precocious Ro expression [38]. *h*^*H10*^ results from a *pGawB* insertion in the *hairy* gene, which is used to drive *hairy*-dependent expression of GAL4 anterior to and within the MF [52]. UAS-mδ was made from the insertion of an EcoRI-XhoI fragment of *E(spl)mδ* cDNA into pUAST for forced-expression [33, 53]. *sens*^*E2*^ is a missense mutant that results in premature translational termination [54]. *ato*^*1*^ encodes A25T, K253N, N261I, the last of which ablates DNA binding [1].

### Immunohistochemistry

All steps were performed at room temperature unless otherwise indicated. Tissues were dissected in 0.1M sodium phosphate buffer and fixed in 4–6% formaldehyde, 0.1M sodium phosphate buffer. Tissues were washed in 0.3% Triton, 0.1M sodium phosphate buffer, 1% BSA, then blocked in 1% BSA, 0.1M sodium phosphate buffer, and incubated in primary antibody mixtures (antibody concentrations shown below in 0.1M sodium phosphate buffer) for 12–18 hours at 4°C. Following primary antibody incubation, tissues were washed in 0.1M sodium phosphate buffer and bathed in secondary antibody mixtures (1:1000 dilution for each secondary, in 1% BSA, 0.1M sodium phosphate buffer) for 2 hrs. Secondary antibody mixtures were removed, tissues were washed 0.1M sodium phosphate buffer. Tissues were mounted in 60% glycerol and imaged using an Olympus Fluoview FV1000 Confocal microscope. All scanning data reported was observed in a minimum of tissues from 5 independent animals of like genotype.

Primary antibodies include rabbit α-Ato (1:5000, [1]); guinea pig α-Sens (1:500–800, [3]); mouse α-E(spl)-mAb323 (1:3, [8]); mouse α-β-gal-40-1a (1:800–1000); rat α-Ciact-2A1 (1:100, [55]); rat α-Elav-7E8A10 (1:100). mouse α-β-gal-40-1a, rat α-Ciact-2A1 and rat α-Elav-7E8A10 were obtained from the Developmental Studies Hybridoma Bank, created by the NICHD of the NIH and maintained at The University of Iowa, Department of Biology, Iowa City, IA 52242. Tissues to be labeled with primary rabbit α-Ato were dissected in 0.3% Triton, 0.1M sodium phosphate buffer.

Secondary antibodies used include 488-goat α-mouse (Jackson), 488-rabbit α-GFP (Life Technologies), 488-goat α-Rat (Life Technologies), 546-goat α-rabbit (Life Technologies), 546-goat α-mouse (Life Technologies), 546-goat α-Rat (Life Technologies), 633-goat α-guinea pig (Life Technologies).

### Light microscopy

Adult/pharate flies were mounted and promptly imaged using a Nikon camera in conjunction with a Leica MZ16 stereomicroscope, and eye size quantified as described [56]. For counts listed as “700+”, facet count exceeded 700. Statistical significance was determined using Student’s T-Test.

### Image production

All images were processed in Adobe Photoshop CC v. 14.2. Image manipulations of brightness/contrast and color balance were applied uniformly across each image shown. Images were then organized in Adobe Illustrator CC v. 17.1.
